# The effect of evening primrose oil on adolescent girl patients with PCOS: A double-blind placebo-controlled randomized study

**DOI:** 10.22038/AJP.2024.24342

**Published:** 2024

**Authors:** Laila Mohammadlo, Kaveh Rahimi, Masomeh Rezaie, Nasrin Soufizadeh, Fariba Seyedoshohadaei, Khaled Rahmani, Reza Bekhradi

**Affiliations:** 1 *Department of Obstetrics and Gynecology, Faculty of Medicine, Kurdistan University of Medical Sciences, Sanandaj, Iran*; 2 *Department of Basic Sciences, Faculty of Veterinary Medicine, Shahid Chamran University of Ahvaz, Ahvaz, Iran*; 3 *Liver and Digestive Research Center, Research Institute for Health Development, Kurdistan University of Medical Sciences, Sanandaj, Iran*; 4 *Barij Medicinal Plants Research Center, Kashan, Iran*

**Keywords:** Evening primrose oil, PCOS, Insulin, Testosterone, FAI, SHBG

## Abstract

**Objective:**

Polycystic ovary syndrome (PCOS) is a common disorder among women of reproductive age. The goal of the current study was to evaluate the effect of evening primrose oil (EPO) (Oenothera biennis) in adolescent girls with PCOS.

**Materials and Methods:**

In the current double-blind and randomized controlled research, 76 patients completed the study in two groups (38 in the placebo and 38 in the EPO groups). The patients were asked to take oral placebo or EPO (1000 mg/day) for 12 weeks. Biochemical, clinical, and ultrasonography assessments were performed. The data was analyzed using SPSS statistical software.

**Results:**

After the intervention, the regulation of the menstrual cycle in the EPO group was different from that of the placebo group (p=0.04). The levels of insulin, testosterone, and free androgen index (FAI) in the EPO group were lower than those of the placebo group (p<0.05). The sex hormone binding globulin (SHBG) levels in the EPO group were higher than those of the placebo group (p=0.01). While the number of immature follicles decreased in the EPO group, this difference was not statistically significant (p=0.8).

**Conclusion:**

Overall, EPO administration for 12 weeks in young women with PCOS regulated the irregular menstrual cycle. In addition, the levels of insulin, testosterone, FAI, and SHBG changed. Therefore, EPO may be effective in improving hormonal and menstrual irregularities.

## Introduction

Polycystic ovarian syndrome (PCOS) is a hormonal disorder in women. In total, 5 to 10% of women are affected by PCOS in their reproductive age. It is worth mentioning that based on the Rotterdam criteria, the prevalence of PCOS in Iran is 15.2% (Kamboj and Bonny, 2017; Kazem Moslemi and Yazdani, 2010; Behboodi Moghadam et al., 2018; Amini et al., 2020). The problems and complications of PCOS include acne, and obesity (Beltadze and Barbakadze, 2015). PCOS is usually accompanied by changes in serum hormone levels. Ovulation is disrupted in women with PCOS because the number of follicles that mature is small. In PCOS, the levels of progesterone, estrogen, luteinizing hormone (LH), and follicle stimulating hormone (FSH) also change. Androgen levels are higher than normal, while progesterone levels are lower (De Leo et al., 2016). Menstrual irregularities and hirsutism are other complications of cystic ovaries (Gupta et al., 2016). Irregular menstrual cycles are related with higher androgen levels and lower sex hormone binding globulin (SHBG) in PCOS patients (Harris et al., 2017).

Currently, drugs such as clomiphene citrate, aromatase inhibitors, tamoxifen, and metformin are used to treat PCOS (Badawy and Elnashar, 2011). The use of medicinal plants as adjuvants along with chemical drugs is effective in treating certain gynecological diseases (Balamurugan et al., 2017). Evening primrose oil (EPO) (Oenothera biennis) contains α-linoleic acid (60 to 80%) and gamma-linoleic acid (8 to 14%) (Bayles and Usatine, 2009). The beneficial effects of EPO may be due to the high levels of essential fatty acids in it which affect the immune system (Vassilopoulos et al., 1997; Sergeant et al., 2016). The efficacy of EPO in the management of different diseases such as mastalgia, premenstrual syndrome, atopic eczema, multiple sclerosis, diabetic neuropathy, rheumatoid arthritis, coronary heart disease, gastrointestinal disorders, renal disease, infections, endometriosis, alcoholism, and dementia has been shown (Kerscher and Korting, 1992; Joe and Hart, 1993; Belch and Hill, 2000; Joy et al., 2000; Morse and Clough, 2006; Stuart, 2014; Bamford et al., 2013). One of the complications of ovarian cyst is infertility, so its treatment is more important in girls who are not yet married. The goal of the current study was to evaluate the effect of EPO on single young women with PCOS.

## Materials and Methods

### Type of study

This randomized double-blind placebo clinical trial was conducted at Sanandaj University of Medical Sciences, Sanandaj, Iran. The registration number of this clinical trial at the Iranian Registry of Clinical Trials is IRCT20200722048175N1. The current study was approved by the Ethics Committee of Sanandaj University of Medical Sciences, Sanandaj, Iran.

### Participants

The participants were single girls between the ages of 18 and 24 years with polycystic ovary syndrome. The study protocol was explained to the patients and written consent was obtained from all participants at the beginning of the study. This study was conducted on patients referred to the obstetrics and gynecology clinic of Besat Sanandaj Hospital.

### Sample size

The minimum sample size was calculated using the following formula: 

n= (z1-α/2+z1-β) 2[σ12+ σ22]/ (µ1-µ2)2 (α=5, β=1) (Kim, 2016).

Also, the random allocation method was based on quadratic random blocking (numbered containers). Random allocation sequence as well as registration of participants was done by a statistical consultant and interventions were assigned to participants by a gynecologist. The sample size was calculated and 35 participants were assigned to each group. To increase the accuracy, 40 participants in each group were finally included.

### Exclusion criteria

The participates were enrolled according to the Rotterdam diagnostic criteria for PCOS (Smet and McLennan, 2018). The use of any medications to treat PCOS, endocrine disorders that are not caused by PCOS, history of liver or renal dysfunction, and any allergy to herbal medicine were considered as the exclusion criteria.

### Intervention

Placebo capsules (sunflower oil) (Naeimi et al., 2020) and Evening primrose oil were supplied by Barij Essence Company (Isfahan, Iran) (https://barijessence.com/en/product/epo/).

The patients were asked to take the drug (1000 mg daily at a specified time) for 12 weeks. The packages containing the drug were coded from 1 to 80. Treatment compliance was evaluated through volunteer-reported pill count and telephone follow-up. The patients received a package at random. The researchers did not know which capsule was EPO or placebo. The company provided a list that indicated which package was assigned to each EPO or placebo capsule. In the end, only the statistician who performed the statistical analysis received the list. The clinical indicators were measured and the biochemical evaluations and ovarian ultrasound (done by a radiologist) were performed before and after the intervention.

### Measurements

Hirsutism was evaluated using the Ferriman-Gallwey method (Lumezi et al., 2018) before and after the intervention. For each of the nine androgen-sensitive areas in the body, a score from 0 (hairless) to 4 (completely masculine) was considered. The total score of hirsutism in patient was considered the sum of these 9 points. To check the regularity or irregularity of the menstrual cycle, the duration of menstrual cycle was measured according to the patient’s report. Intravenous blood samples were taken from the patients and serum separation was done to evaluate the testosterone, SHBG, and insulin levels. Hormonal studies were performed using ELISA methods. A radiologist who was not aware of the allocations evaluated the ovarian volume and determined the number of follicles using ultrasound. The free androgen index (FAI) was employed to determine abnormal androgen status using total testosterone levels divided by sex hormone binding globulin (SHBG) levels and multiplied by 100.

### Data analysis

The data were analyzed using the SPSS (version 22). Data analysis was done using one-way ANOVA statistical method. The quantitative variables are presented as mean±standard deviation. In comparing the two groups, for categorical data the chi-square test was used and the Student’s t-test was employed for continuous parameters. Univariate analysis of covariance (ANCOVA) test was applied to disclose any differences between two intervention groups at the end of trial. A p<0.05 was considered significant. 

## Results

### Patients’ information

**Figure 1:**
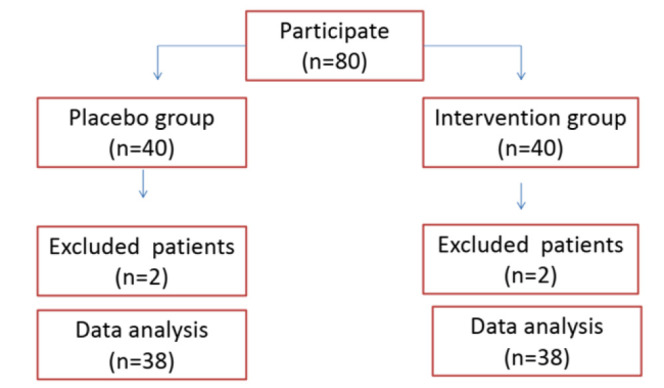
Flowchart of the study

Eighty patients entered the study (40 in the placebo and 40 in the EPO groups). However, seventy-six patients were present in our study until the end of the intervention (38 in the placebo and 38 in the EPO groups) (Figure 1). Four patients were excluded due to non-cooperation. In the current study, patients were homogeneous in terms of demographic characteristics. The mean ages in the placebo and EPO groups were 21.29±2.61 and 21.29±2.99 years, respectively (p=0.9). 

### Patient evaluation data

The body mass index (BMI) before the intervention was not significantly different between the placebo and EPO groups (p=0.92). The BMI after the intervention was not significantly different in the EPO group compared with the placebo group (p=0.98) (Tables 1 and 2). 

Before the intervention the degree of hirsutism was not significantly different between the placebo and EPO groups (p=0.66). The rate of hirsutism after the intervention was not reduced statistically significantly in the EPO group compared with the placebo group (p=0.22) (Tables 1 and 2). 

The number of follicles before the intervention was not significantly different between the placebo and EPO groups (p=0.20). The number of follicles after the intervention was not statistically significantly different between the EPO and the placebo group (p=0.21) (Tables 1 and 2). 

The volume of the ovary before the intervention was not significantly different between the placebo and EPO groups (p=0.59). The volume of the ovary after the intervention was not reduced statistically significantly in the EPO group compared with the placebo group (p=0.80) (Tables 1 and 2).

The irregular menstrual cycle before the intervention was not different between the study groups. Thus, in the placebo group, the regular menstrual cycle was 7.9% and the irregular menstrual cycle was 92.1%. In the intervention group, the regular menstrual cycle was 2.6% and the irregular menstrual cycle was 97.4%. Regulatory of the menstrual cycle after the intervention was statistically significantly different in the EPO group compared with the placebo group. Thus, in the placebo group, the regular menstrual cycle was 13.2% and the irregular menstrual cycle was 86.8%. In the intervention group, the regular menstrual cycle was 42.1% and the irregular menstrual cycle was 57.9% (Tables 3). 

### Biochemical measurements

**Figure 2:**
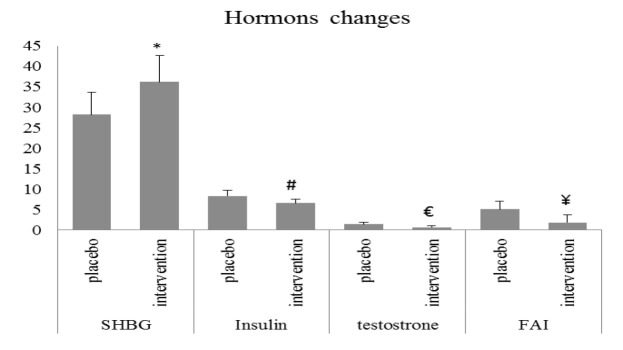
Hormone changes in different groups. Group 1: placebo. Group 2: EPO 1000 mg/day. Independent Samples Test. P value less than 5% was considered significant. *, #, €, and ¥ show a significant difference between the intervention group and the placebo group for each parameter. (Group 1; placebo, Group 2: intervention). SHBG:sex hormone binding glubolin, FAI: free androgen index.

After the intervention, the levels of insulin, testosterone, sex hormone binding globulin (SHBG), and FAI were statistically significantly different in the EPO group compared with the placebo group. The insulin levels in the EPO group were lower than those of the placebo group (p=0.01). The SHBG levels in the EPO group were higher than those of the placebo group (p=0.04). The testosterone levels in the EPO group were lower than those of the placebo group (p=0.03). The FAI levels in the EPO group were lower than those of the placebo group (p=0.03) (Figure 2). No side effects related to drug use were observed.

## Discussion

It was demonstrated that the administration of EPO for twelve weeks in patients with PCOS significantly improved the regulation of the menstrual cycle. In addition, the insulin, SHBG, testosterone, and FAI levels after the intervention were statistically significantly different in the EPO group compared to the placebo group. 

PCOS leads to various disorders in girls including menstrual irregularities, hirsutism, and infertility. It also leads to changes in endocrine hormones (low levels of progesterone and high levels of luteinizing hormone, testosterone, estrogen, and prolactin) and metabolic disorders  such as dyslipidemia, obesity, inflammation, and high blood pressure (Abasian et al., 2018).

Our data showed that the daily use of EPO resulted in regular menstruation in patients with PCOS. Irregular menstrual cycles are associated with higher androgen and lower SHBG in patients with PCOS. Therefore, changes in the hormonal profile by EPO may lead to a regular menstrual cycle in patients. Previous studies have shown that EPO in rats with estradiol-induced PCOS reduces the levels of testosterone and LH and increases the levels of FSH (Zand Vakili et al., 2018).

Insulin resistance leads to elevated free androgen levels and thus a decrease in follicular growth in PCOS (Ebrahimi-Mamaghani et al., 2015). Decreased serum SHBG levels in PCOS are due to elevated insulin levels that increase testosterone levels. In addition, these patients have abnormal gonadotropin concentrations due to high insulin levels (Zeng et al., 2020). In a related study, EPO increased insulin sensitivity in rats with PCOS (Zand Vakili et al., 2018).

Antioxidant and anti-inflammatory compounds increase the number of growing follicles (Bardei, 2015). Our data demonstrated that EPO can reduce testosterone levels in the intervention group compared to the placebo group. Excessive secretion of adrenal precursor androgen has been reported in PCOS (Sadoughi, 2017). Hyperandrogenism may be involved in the development of metabolic disorders in patients with PCOS. SHBG is involved in the regulation of sex steroid hormone function and low levels of SHBG are associated with an increased risk of PCOS. Patients with PCOS and low SHBG levels are prone to obesity, hyperandrogenism, insulin resistance, and infertility (Hopkinson et al., 1998; Deswal et al., 2018). The FAI increases in PCOS. As a discriminator of PCOS, FAI seems to be more accurate in patients younger than forty years of age (Pinola et al., 2015). In our results, the SHBG levels in the EPO group were higher than those of the placebo group. In addition, the FAI levels in the EPO group were lower than those of the placebo group. 

Various studies suggest that herbal medicines may play a role in the treatment of PCOS (Arentz et al., 2014; Abasian et al., 2018). These herbs improve the conditions of PCOS patients by changing the estrogen receptor (Radha et al., 2014; Gholamalizadeh et al., 2018). The effects of EPO are probably related to gamma-linolenic acid (γ-LA) which can lead to anti-inflammatory effects (Belch and Hill, 2000). The analysis of EPO showed that it consists of 74% LA, 7% oleic, and 9% γ-LA and that its beneficial properties are due to the effects of these essential fatty acids (Fan and Chapkin, 1998). Using EPO as a supplement increases the levels of γ-LA (9.24%) as well as its metabolite (dihomo-γ-LA) in blood plasma. γ-LA is oxidized by 5-lipoxygenase to 15-hydroxyeicosatrienoic acid (15-HETrE) and dihomo-γ-LA is converted by cyclooxygenase to series 1 prostaglandins. These compounds have anti-inflammatory properties (Timoszuk et al., 2018). Using EPO seems to be effective in the management of various disorders in women (mastalgia, premenstrual syndrome, cervical ripening gestational diabetes, fibroadenomas, and endometriosis) (Mahboubi, 2019). 

 The histomorphometric examination of the ovarian tissue in rats with letrozole-induced PCOS showed that EPO increased corpus luteum and decreased cystic follicles compared with the controls. This study suggested that EPO could have beneficial effects on PCOS by improving folliculogenesis (Zand Vakili et al., 2018). Herbal medicines, such as *Cinnamomum*, fennel, *Cimicifuga racemosa*, * Vitex agnus-castus*,  and *Tribulus terrestris*, *Glycyrrhiza *spp have been able to reduce the complications of PCOS by improving the hormonal profile (Arentz et al., 2014).

Considering that this study was conducted on young unmarried girls, the limitations of this study included the reluctance of the patients to use herbal medicines and it took a lot of time to reach the desired number of samples.

Natural compounds (such as EPO) on which sufficient studies have been conducted can be used as a therapeutic supplement. The current study for the first time shows that EPO can be useful as a therapy for the PCOS patients by improvement of steroid hormone synthesis. 

**Table 1:** Demographical information of the study groups before intervention

	**Group **	**N**	**Mean**	**Std. Deviation**	**Sig **
**Body mass index (BMI) [kg/m2, median (range)]** **before intervention**	placebo	38	24.69	2.61	0.92
	intervention	38	24.75	2.99	
**Age (year)**	placebo	38	22.29	4.28	0.12
	intervention	38	21.29	4.45	
**Hirsutism before intervention **	placebo	38	9.24	6.24	0.45
	intervention	38	10.39	7.13	
**Number of follicles before intervention**	placebo	38	15.95	3.46	1.00
	intervention	38	15.95	2.59	
**Ovarian volume before intervention**	placebo	38	14.03	4.38	0.97
	intervention	38	14.00	3.86	

**Table 2:** Information of the patients after the intervention.

	**group**	**N**	**Mean**	**Std. Deviation**	**Sig **
**body mass index (BMI) [kg/m2, median (range)] after intervention**	placebo	38	24.89	4.16	0.98
	intervention	38	24.72	4.37	
**Hirsutism after intervention **	placebo	38	9.29	5.94	0.22
	intervention	38	7.21	5.03	
**Number of follicles after intervention**	placebo	38	15.18	3.33	0.21
	intervention	38	13.76	3.83	
**Ovarian volume after intervention**	placebo	38	14.34	5.31	0.80
	intervention	38	12.42	4.47	

Group 1: placebo. Group 2: EPO 1000 mg/day. Independent Samples Test. p value less than 5% was considered significant

**Table 3:** Menstrual cycle status before the intervention

** Menstrual before**					
			**Regular**	**Irregular**	**Total**
group	placebo	Count	3	35	38
		% within group	7.9%	92.1%	100.0%
	intervention	Count	1	37	38
		% within group	2.6%	97.4%	100.0%
Total		Count	4	72	76
		% within group	5.3%	94.7%	100.0%
**Menstrual after**					
group	placebo	Count	5	33	38
		% within group	13.2%	86.8%	100.0%
	intervention	Count	16	22	38
		% within group	42.1%	57.9%	100.0%
Total		Count	21	55	76
		% within group	27.6%	72.4%	100.0%

Group 1: placebo. Group 2: EPO 1000 mg/day. Chi-Square Tests. p value less than 5% was considered significant.

## References

[B1] Abasian Z, Rostamzadeh A, Mohammadi M, Hosseini M, Rafieian-kopaei M (2018). A review on role of medicinal plants in polycystic ovarian syndrome: Pathophysiology, neuroendocrine signaling, therapeutic status and future prospects. Middle East Fertil Soc J.

[B2] Amini L, Mojab F, Jahanfar S, Sepidarkish M, Raoofi Z, Maleki-Hajiagha A (2020). Efficacy of Salvia officinalis extract on the prevention of insulin resistance in euglycemic patients with polycystic ovary syndrome: A double-blinded placebo-controlled clinical trial. Complement Ther Med.

[B3] Arentz S, Abbott JA, Smith CA, Bensoussan A (2014). Herbal medicine for the management of polycystic ovary syndrome (PCOS) and associated oligo/amenorrhoea and hyperandrogenism; a review of the laboratory evidence for effects with corroborative clinical findings. BMC Complement Altern Med.

[B4] Badawy A, Elnashar A (2011). Treatment options for polycystic ovary syndrome. Int J Womens Health.

[B5] Balamurugan S, Vijayakumar S, Prabhu S, Morvin Yabesh JE (2017). Traditional plants used for the treatment of gynaecological disorders in Vedaranyam taluk, South India - An ethnomedicinal survey. J Tradit Complement Med.

[B6] Bamford JT, Ray S, Musekiwa A, van Gool C, Humphreys R, Ernst E (2013). Oral evening primrose oil and borage oil for eczema. Cochrane Database Syst Rev.

[B7] Bardei K (2015). The effects of hydro-alcoholic extract of raspberry fruit on ovarian follicles and serum parameters in poly cystic ovary syndrome-induced rat. Armaghane Danesh.

[B8] Bayles B, Usatine R (2009). Evening primrose oil. Am Fam Physician.

[B9] Behboodi Moghadam Z, Fereidooni B, Saffari M, Montazeri A (2018). Polycystic ovary syndrome and its impact on Iranian women’s quality of life: a population-based study. BMC Women's Health.

[B10] Belch J, Hill A (2000). Evening primrose oil and borage oil in rheumatologic conditions. Am J Clin Nutr.

[B11] Beltadze K, Barbakadze L (2015). Diagnostic features of polycystic ovary syndrome in adolescents (review). Georgian Med News.

[B12] De Leo V, Musacchio MC, Cappelli V, Massaro MG, Morgante G, Petraglia F (2016). Genetic, hormonal and metabolic aspects of PCOS: an update. Reprod Biol Endocrinol.

[B13] Deswal R, Yadav A, Dang AS (2018). Sex hormone binding globulin-an important biomarker for predicting PCOS risk: A systematic review and meta-analysis. Syst Biol Reprod Med.

[B14] Ebrahimi-Mamaghani M, Saghafi-Asl M, Pirouzpanah S, Aliasgharzadeh A, Aliashrafi S, Rezayi N, Mehrzad-Sadaghiani M (2015). Association of insulin resistance with lipid profile, metabolic syndrome, and hormonal aberrations in overweight or obese women with polycystic ovary syndrome. J Health Popul Nutr.

[B15] Fan Y-Y, Chapkin RS (1998). Importance of dietary γ-linolenic acid in human health and nutrition. J Nutr.

[B16] Gholamalizadeh M, Doaei S, Akbari ME, Rezaei S, Jarrahi AM (2018). Influence of fat mass-and obesity-associated genotype, body mass index, and dietary intake on effects of iroquois-related homeobox 3 gene on body weight. Chin Med J (Engl).

[B17] Gupta S, Pandithurai E, Agarwal A, Kumanov, P, Agarwal, A (2016). Polycystic ovary syndrome in adolescent girls.

[B18] Harris HR, Titus LJ, Cramer DW, Terry KL (2017). Long and irregular menstrual cycles, polycystic ovary syndrome, and ovarian cancer risk in a population-based case-control study. Int J Cancer.

[B19] Hopkinson ZE, Sattar N, Fleming R, Greer IA (1998). Polycystic ovarian syndrome: the metabolic syndrome comes to gynaecology. BMJ.

[B20] Joe LA, Hart LL (1993). Evening primrose oil in rheumatoid arthritis. Ann Pharmacother.

[B21] Joy C, Mumby-Croft R, Joy L (2000). Polyunsaturated fatty acid (fish or evening primrose oil) for schizophrenia. Cochrane Database Syst Rev.

[B22] Kamboj MK, Bonny AE (2017). Polycystic ovary syndrome in adolescence: diagnostic and therapeutic strategies. Transl Pediatr.

[B23] Kazem Moslemi M, Yazdani Z (2010). A huge ovarian cyst in a middle-aged Iranian female. Case Rep Oncol.

[B24] Kerscher M, Korting H (1992). Treatment of atopic eczema with evening primrose oil: rationale and clinical results. J Clin Investig.

[B25] Kim HY (2016). Statistical notes for clinical researchers: Sample size calculation 1 comparison of two independent sample means. Restor Dent Endod.

[B26] Lumezi BG, Berisha VL, Pupovci HL, Goçi A, Hajrushi AB (2018). Grading of hirsutism based on the Ferriman-Gallwey scoring system in Kosovar women. Postepy Dermatol Alergol.

[B27] Mahboubi M (2019). Evening primrose (Oenothera biennis) oil in management of female ailments. J Menopausal Med.

[B28] Morse N, Clough P (2006). A meta-analysis of randomized, placebo-controlled clinical trials of Efamol® evening primrose oil in atopic eczema Where do we go from here in light of more recent discoveries?. Curr Phar Biotechnol.

[B29] Naeimi SA, Tansaz M, Hajimehdipoor H, Saber S (2020). Comparing the effect of Nigella sativa oil soft gel and placebo on oligomenorrhea, amenorrhea and laboratory characteristics in patients with polycystic ovarian syndrome, a randomized clinical trial. RJP.

[B30] Pinola P, Piltonen TT, Puurunen J, Vanky E, Sundström-Poromaa I, Stener-Victorin E, Ruokonen A, Puukka K, Tapanainen JS, Morin-Papunen LC (2015). Androgen profile through life in women with polycystic ovary syndrome: A nordic multicenter collaboration study. J Clin Endocrinol Metab.

[B31] Radha M, Padamnabhi N, Laxmipriya N (2014). Evaluation of Aloe barbadensis mill Gel on letrozole induced polycystic ovarian syndrome (pcos) rat model-a dose dependent study. IJPSR.

[B32] Sadoughi S (2017). Effects of crocin on ovarian follicle and serum sex hormone in letrozole-induced polycystic ovarian syndrome in rat model. J Ardabil Univ Med Sci.

[B33] Sergeant S, Rahbar E, Chilton FH (2016). Gamma-linolenic acid, Dihommo-gamma linolenic, Eicosanoids and Inflammatory Processes. Eur J Pharmacol.

[B34] Smet M-E, McLennan A (2018). Rotterdam criteria, the end. Australas J Ultrasound Med.

[B35] Stuart J (2014). Herbal Medicines Fourth edition. J Med Libr Assoc.

[B36] Timoszuk M, Bielawska K, Skrzydlewska E (2018). Evening primrose (Oenothera biennis) biological activity dependent on chemical composition. Antioxidants.

[B37] Vassilopoulos D, Zurier RB, Rossetti RG, Tsokos GC (1997). Gammalinolenic acid and dihomogammalinolenic acid suppress the CD3-mediated signal transduction pathway in human T cells. Clin Immunol Immunopathol.

[B38] Zand Vakili F, Zare S, Rahimi K, Riahi M (2018). The effect of evening primrose oil on changes in polycystic ovary syndrome induced by estradiol valerate in rat. Armaghane Danesh.

[B39] Zeng X, Xie Y-j, Liu Y-t, Long S-l, Mo Z-c (2020). Polycystic ovarian syndrome: correlation between hyperandrogenism, insulin resistance and obesity. Clinica Chimica Acta.

